# Changes in the theta band coherence during motor task after hand immobilization

**DOI:** 10.1186/1755-7682-7-51

**Published:** 2014-12-13

**Authors:** Igor Brauns, Silmar Teixeira, Bruna Velasques, Juliana Bittencourt, Sergio Machado, Mauricio Cagy, Mariana Gongora, Victor Hugo Bastos, Dionis Machado, Ada Sandoval-Carrillo, Jose Salas-Pacheco, Roberto Piedade, Pedro Ribeiro, Oscar Arias-Carrión

**Affiliations:** Brain Mapping and Sensory Motor Integration, Institute of Psychiatry of Federal University of Rio de Janeiro (IPUB/UFRJ), Rio de Janeiro, Brazil; School of Physical Education, Bioscience Department (EEFD/UFRJ), Rio de Janeiro, Brazil; Biomedical Engineering Program, COPPE, Federal University of Rio de Janeiro, Rio de Janeiro, Brazil; Institute of Applied Neuroscience (INA), Rio de Janeiro, Brazil; National Institute of Traumatology and Orthopaedics (INTO), Neuromuscular Research Laboratory, Rio de Janeiro, Brazil; Instituto de Investigación Científica, Universidad Juárez del Estado de Durango, Durango, Durango, México; Institute of Psychiatry of Federal University of Rio de Janeiro, Panic and Respiration, Rio de Janeiro, Brazil; National Institute for Translational Medicine (INCT-TM), Rio de Janeiro, Brazil; Physical Activity Neuroscience, Physical Activity Sciences Postgraduate Program, Salgado de Oliveira University, Niterói, Brazil; Brain Mapping and Functionality Laboratory, Federal University of Piauí, UFPI, Parnaiba, Brazil; Brain Mapping and Plasticity Laboratory, Federal University of Piauí, UFPI, Parnaiba, Brazil; Unidad de Trastornos del Movimiento y Sueño (TMS), Hospital General Dr. Manuel Gea González/IFC-UNAM, Mexico City, Mexico; Unidad de Trastornos del Movimiento y Sueño (TMS), Hospital General Ajusco Medio, Secretaria de Salud Mexico City, Mexico

**Keywords:** Hand immobilization, qEEG coherence, Sensorimotor integration, Theta band

## Abstract

Many different factors can temporarily or permanently impair movement and impairs cortical organization, e.g. hand immobilization. Such changes have been widely studied using electroencephalography. Within this context, we have investigated the immobilization effects through the theta band coherence analysis, in order to find out whether the immobilization period causes any changes in the inter and intra-hemispheric coherence within the cerebral cortex, as well as to observe whether the theta band provides any information about the neural mechanisms involved during the motor act. We analyzed the cortical changes that occurred after 48 hours of hand immobilization. The theta band coherence was study through electroencephalography in 30 healthy subjects, divided into two groups (control and experimental). Within both groups, the subjects executed a task involving flexion and extension of the index finger, before and after 48 hours. The experimental group, however, was actually submitted to hand immobilization. We were able to observe an increase in the coupling within the experimental group in the frontal, parietal and temporal regions, and a decrease in the motor area. In order to execute manual tasks after some time of movement restriction, greater coherence is present in areas related to attention, movement preparation and sensorimotor integration processes. These results may contribute to a detailed assessment of involved neurophysiological mechanism in motor act execution.

## Introduction

Sensorimotor integration mechanisms can be affected by many factors, among which are hand-immobilizing lesions that can temporarily or permanently impair movement and bring about changes to the cortical organization [1, 2] in addition to provoking a reduction of neuromuscular activity [3]. This has been observed in the study by Gondin [4] after submitting subjects to a two-week immobilization. Liepert [5] have also highlighted such phenomenon after submitting individuals to movement restriction. In addition to this, Seki [6] have found a reduction in motor neuron firing after a week of finger immobilization. In fact, Facchini [7] have observed that the cortical and neuromuscular excitability indexes are not influenced by a four-day period of finger immobilization. Within this context, body limbs immobilization has been used in many studies, not only to verify neuromuscular changes, but also to understand the influence of sensorimotor restriction on cortical behavior [6, 8–10].

Among the tools used to investigate the cortical changes after an immobilization period, quantitative electroencephalography (qEEG) has been widely employed [11, 12] and it has provided information about cortical activity with high temporal definition in motor, sensory and cognitive tasks [13, 14]. In particular, the qEEG theta band (4–8 Hz) has been associated to cognitive processing [15, 16] and to sensory stimuli identification and codification [17–19]. This way, theta band oscillations have been related to sensorimotor integration mechanisms [20]. In particular, the coherence analysis provides evidence of the coupling between cortical areas during task execution [21, 22]. Although researches have demonstrated the theta band involvement in studies about coherence in cognitive, sensory and motor tasks [17, 20], little is known about its activity when related to movement restriction.

This way, our study aimed at analyzing theta band coherence after 48 hours of hand immobilization. The electrode derivations were selected through qEEG in different brain regions: the frontal region, due to its relationship with the pre-motor and pre-frontal cortexes, which are functionally related to action creation and voluntary control [23] and to executive functions [24]; the central region, which represents motor act execution [25, 26]; the parietal region, because of its relationship with sensorimotor integration [23, 27] and the temporal region, since it represents the secondary motor areas [28]. Therefore, our study hypothesized that the hand immobilization period provokes changes in the inter and intra-hemispheric coherence in the frontal, central, parietal and temporal regions, as well as providing information about the neural mechanisms involved in the motor act execution. The objective of the present study is then to analyze the theta band coherence oscillations after 48 hours of hand immobilization.

## Methods

### Sample

The experimental group sample was composed of 15 healthy individuals (07 men and 08 women; mean age ± standard deviation [SD] = 24 ± 1.2 years; age range = 20–30 years); the control group sample was composed of 15 healthy individuals (07 men and 08 women; mean age ± SD 23 ± 1.4 years; age range = 20–30 years). The individuals were chosen randomly and the recruitment of the volunteers was accomplished thanks to the research announcements posted in different universities of Rio de Janeiro State. As inclusion criteria, the subjects needed to be right handed, have no mental or physical illness (previous anamnese) and not use any psychoactive or psychotropic substances during the whole time of the study. We applied a detailed questionnaire in order to exclude those individuals who could contaminate our results. Due to hand laterality, we utilized the Edinburgh inventory [29] to identify the predominance of the participants (right handed vs. left-handed). Consequently, the left-handed individuals were excluded from the experiment. We instructed the individuals to not use tobacco, coffee or alcoholic drinks 10 hours before the test. The participants received written information about the study procedures and we solicited their signature of the consent form. This study was approved by the ethics committee of Veiga de Almeida University with the number 149.817 in accordance with the ethical standards laid down in the 1964 Declaration of Helsinki.

### Tasks and procedures

We prepared a room with acoustic and electrical isolation. During the electroencephalography (EEG) signal acquisition, the lights were dimmed. The subjects sat in a chair with armrest, in order to minimize muscle artifact during EEG signal acquisition. A 15-inch monitor was placed on a table in front of the subjects. The monitor was turned on only when the subjects executed the task (i.e., flexion and extension of the index finger). Initially, the EEG signal acquisition lasted for 2 minutes (rest) with the monitor off, facing the subjects. Then, we coupled a sensor to measure acceleration (accelerometer) on the right index finger; during the visual feedback, the subjects executed the task (i.e., flexion and extension of the index finger). The accelerometer was connected to the EEG through an additional channel (i.e., channel 21). When the subjects performed the movement, the accelerometer provided a signal for the EEG. The subjects were instructed to perform the index finger flexion and extension when visual feedback was generated by a random image on the monitor. The subjects executed the task in 6 blocks of 15 trials. In order to avoid muscle fatigue, they rested for 3 minutes between each block. Thus, the task lasted 1 minute for each block with a 3 minute-interval between blocks, totaling 24 minutes for the whole task. After completing the task, the monitor was turned off and the subjects were submitted again to EEG during 2 minutes (rest). After EEG recording, a plaster cast was applied on the subjects’ right hand and they kept it on for 48 hours. The plaster cast was applied with the hand closed, in order to prevent any hand or finger movement. After this period, the experimental group subjects returned to the laboratory to remove the plaster cast and, after five minutes from cast removal, they were submitted again to the same task procedures executed before hand immobilization. The control group (non-plaster cast), after EEG recording, went back to the laboratory after 48 hours and executed the procedures again.

### Data acquisition

#### Electroencephalography

The International 10/20 system for electrodes was used with a 20-channel Braintech-3000 EEG system (EMSA-Medical Instruments, Brazil). The 20 electrodes were arranged in a nylon cap (ElectroCap Inc., Fairfax, VA, USA), yielding mono-pole derivations to linked earlobes. In addition, two 9-mm diameter electrodes were attached above and on the external corner of the right eye, in a bipolar electrode montage, to monitor artifacts on eye-movements (EOG). Impedance of EEG and EOG electrodes was kept under 5–10 KΩ. The data acquired had total amplitude of less than 100 μV. The EEG signal was amplified with a gain of 22,000, analogically filtered between 0.01 Hz (high-pass) and 100 Hz (low-pass), and sampled at 240 Hz. The software Data Acquisition (Delphi 5.0) was employed to filter the raw data: notch (60 Hz), high-pass of 0.3 Hz and low-pass of 100Hz.

### Accelerometer

In order to obtain signals from the accelerometer, the MMA7340 model of Freescale semiconductors was used. This system is a microelectronics mechanism, which explores the mechanic proprieties of silicone to create movable structures and to detect distinct movement directions [30]. The movement capture was conducted in actual time system, with the interaction of EEG software signal acquisition. As the movement was performed, the accelerometer showed a curve with acceleration variability providing information about velocity and time.

### Data processing

In order to quantify reference-free data, a visual inspection and independent component analysis (ICA) was applied to identify and remove any remaining artifacts, i.e., eye blinks and ocular movements, produced by the task [31]. Data from individual electrodes exhibiting loss of contact with the scalp or high impedances (>10 kΩ) were discarded, and data from single-trial epochs exhibiting excessive movement artifacts (±100 μV) were also deleted. ICA was then applied to identify and remove any artifacts after the initial visual inspection. ICA is an information maximization algorithm to blind the EEG signals related to the artifacts [31]. Independent components resembling eye-blink or muscle artifacts were removed and the remaining components were then projected back onto the electrode data by multiplying it by the inverse matrix of the spatial filter coefficients derived from ICA, using established procedures. The ICA-filtered data were then reinspected for residual artifacts using the same rejection criteria described above. Then, a classic estimator was applied for the power spectral density, or directly from the square modulus of the Fourier Transform, performed by MATLAB (Matworks, Inc.). Quantitative EEG parameters were reduced to 4 s periods (the selected epoch started 2 s before and ended 2 s after the trigger). For this analysis, the channel 21 created for the accelerometer was excluded, in order to avoid any artifact (previously published by our group [11]).

### Electrode spatial localization

Four regions of interest were selected: the pre-motor and pre-frontal cortex, represented by the F3/F4, F3/Fz and F7/Fz derivations, which are functionally responsible for action creation and voluntary control [24] and for executive functions [25]; the C3/Cz, C3/C4 and C4/Cz derivations for being representatives of primary motor areas related to the motor act execution [26]; the P3/Pz and P3/P4 derivations, due to their relationship with sensorimotor integration [2, 23, 32] and the secondary motor areas, represented by the T3/T4 derivations. For such analysis, theta band (4–7 Hz) was used, and it was chosen because of its association to cognitive functions, such as sensory information codification [33], attention mechanisms [34] and multi-sensory information transmission [13].

### Statistical analysis

The coherence values were analyzed using a two-way ANOVA, with two factors: group (control and experimental) and visit (before and after 48 hours) for each derivation studied (F3/F4, F3/Fz, F7/Fz, C3/Cz, P3/Pz, P3/P4 and T3/T4). All possible interactions were examined by the one-way ANOVA. The significance level was established at p ≤ 0.007 (0.05/7 = 0.007).

## Results

**Figure 1:**
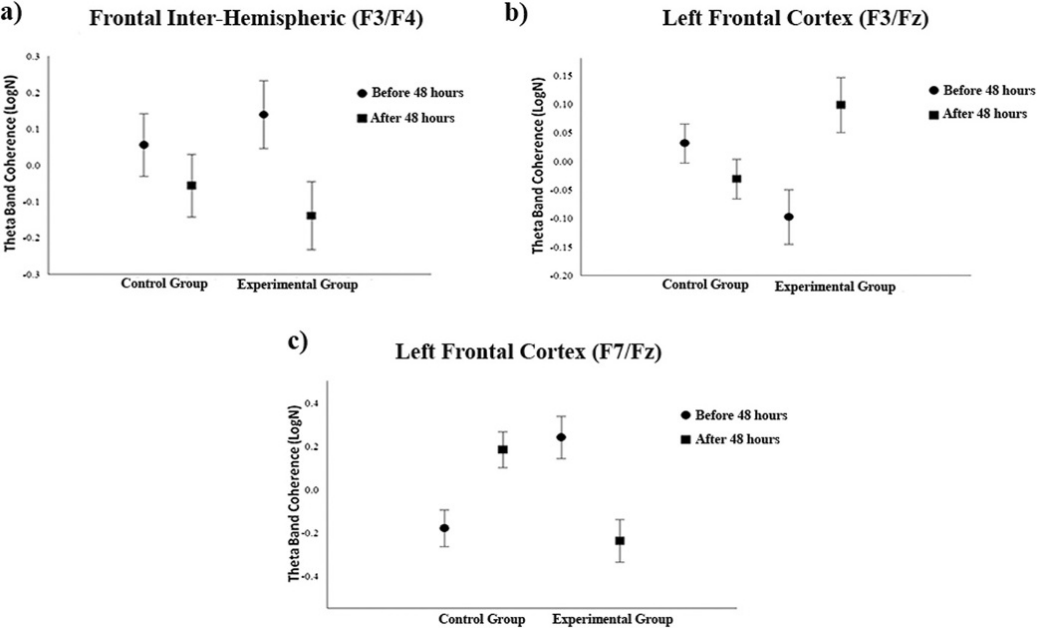
**Cortical changes during motor tasks. a)** Effects for factor group observed in the frontal inter-hemisphere (F3/F4) derivations by mean and SD (p < .001); **b)** Main effects for factor treatment observed in the left frontal cortex (F3/Fz) derivation by mean and SD (p < .001); **c)** Effects for factor treatment observed in the F7/Fz derivations by mean and SD (p = .017).

**Figure 2:**
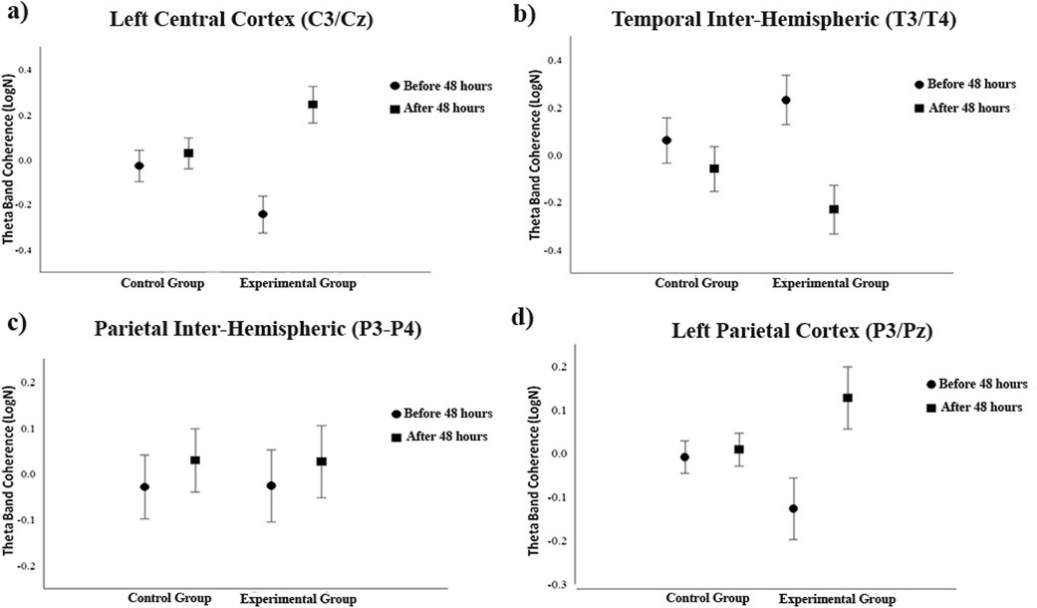
**Cortical changes occurring after hand immobilization. a)** Effects for factor group observed in the left central cortex (C3/Cz) derivations by mean and SD (p = .022); **b)** Effects for factor treatment observed in the temporal inter-hemispheric (T3/T4) derivation by mean and SD (p = .004); **c)** Effects for factor treatment observed in the P3/P4 derivations by mean and SD (p = .004) and **d)** Main effects for factor treatment observed in the left parietal cortex (P3/Pz) derivation by mean and SD (p < .0001).

The data analysis was conducted using a two-way ANOVA with two factors: group (control and experimental) and visit (before and after 48 hours). A main effect was found for the F3/F4 [F (1.162) = 18.798; p = 0.001] derivations, with a coherence decrease in the control group, when compared to the experimental group (Figure 1a). A main effect with coherence decrease was also found for the F3/Fz [F (1.139) = 15.915; p = 0.001] derivations, showing a coherence reduction in the control group (Figure 1b). However, when analyzed within the groups, a main effect was found for the F7/Fz [F (1.80) = 5.992; p = 0.017] derivations (Figure 1c). Such finding shows that 48 hours of immobilization had an influence on the cortical activity between the pre (mean = -1.4448; SD = 0.744190) and post (mean = -1.6838; SD = 0.57597) moments. In addition to this, a main effect was also observed for the C3/Cz [F (1.153) = 5.335; p = 0.022] derivations, in the group condition with a coherence reduction for the experimental group, and a coherence reduction also occurred among the subjects of this group when examining the pre (mean = - 0.1883; SD = 0.64639) and post-immobilization (mean = -0.5445; SD = 0.40699) moments (Figure 2a). For the T3/T4 [F (1.79) = 5,08; p = 0.027] derivations, a main effect was found, but a significant difference was found only in the experimental group, highlighting a coherence reduction between before (mean = 0.1798; SD = 0.1442) and after (mean = 0.1308; SD = 0.0795) the immobilization period (Figure 2b). For the P3/P4 [F (1.155) = 8.651; p = 0.004] derivations, a main effect was observed in the experimental group, with a theta coherence reduction between the pre (mean = -1.9458; SD = 0.47145) and post-immobilization (mean = -1.9197; SD = 0.49664) moments (Figure 2c). In the parietal region, a main effect was found for the experimental group, with a decrease in coherence for the P3/Pz [F (1.114) = 39.903; p = 0.001] derivations, and an increase took place when analyzing the pre (mean = -0.6441; SD = 0.45417) and post (mean = -0.5445; SD = 0.35792) moments (Figure 2d).

## Discussion

The aim of the present study was to analyze the changes in theta band coherence after 48 hours of hand immobilization. In particular, we hypothesized that the theta band coherence oscillations provide information about the neural mechanisms involved in the motor act execution. In addition to this, changes were expected to occur in the inter- and intra-hemispheric coupling in the areas related to motor planning, sensorimotor integration, executive functions and motor act execution after 48 hours of hand immobilization. Our discussion is organized according to cortical regions (i.e., frontal, central, parietal and temporal), whose specific characteristics were related to theta band coherence.

### Theta band coherence and its relationship with attention and movement preparation

Theta band coherence increased in the frontal region, especially for the F3/Fz derivations. Other studies have demonstrated that the pre-motor and pre-frontal cortexes are related to action creation and voluntary control and to executive functions [28, 31], and that theta activity occurs during that behavior which demands planning updating for the motor act, according to the received sensory information [5, 35]. This way, due to the fact of the coherence analysis presenting evidence of the coupling between cortical areas during motor task execution [21, 22], our findings demonstrate that, in order for the individual to execute the task, greater coupling between these areas was needed for the planning and motor response to the stimulus, which then demanded greater joint action of such cortical regions [36]. However, coherence reduction was detected for the F3/F4 derivations in the experimental group. This finding can be associated to the study by Hatfield [37], where they observed that learning is a skill-specific process and that those structures which are not directly responsible for task execution show a reduced activity when practicing the task, while the system directs its activity to areas that are more important for task execution. Therefore, we support the idea that our task does not provide any learning and, consequently, it would not promote changes such as those encountered for the F3/F4 derivations. However, due to the fact of such derivations being related to Broadmann area 8, we believe that their relationship with visuo-motor cortical activity and planning demonstrate greater efficiency for task execution [38]. This way, the immobilization period (i.e., 48 hours) demanded less coupling between the F3/F4 derivations, in order to meet the needs of other cortical regions involved in task execution; in other words, this area used all of its efficiency potential to execute the motor task [35, 39]. Such change in theta has also been observed in the study by Haufler [40] where the authors compared the cortical activity between beginner and professional shooters during a shooting competition. They found that theta band activity during the shooting task was lower in the professional shooters than in the beginners. Such finding complements our interpretation that immobilization caused greater efficiency for these derivations. In addition to this, the concentration and attention involved in motor task planning are lower when considering simple tasks [41–43]. In fact, when looking at the study by Haufler [40], theta band in experienced shooters was lower, due to the great amount of attention and concentration required of these professionals; on the other hand, our task did not promote an increase in attention nor concentration, since such task (i.e., flexion and extension of the index finger) involved simple daily movements. Despite the possible interpretation stating that the visual stimulus presented for task execution would be an indicator of attention increase, our analysis was carried out during the periods before and after the finger movement and, therefore, we cannot make such statement.

### Theta band and motor act

A decrease in theta band coherence for the C3/Cz derivations in the experimental group can be associated with neurophysiologic findings, which relate such derivations to sensorial facilitation, movement [44, 45], motor act execution [25, 46], temporal organization, sequential movement coordination [47] and motor act organization [47, 48]. In addition to this, researches show that, after short periods of hand immobilization, cortical activity decreases in the motor region related to movement restriction [9, 49]. This way, the changes caused by hand immobilization are more significant when looking at the cortical activity decrease than at the changes themselves, which occurred after the motor act [50]. Within this context, our results indicate that the cortical representation of our task’s sequential actions (i.e., flexion and extension of the index finger) was influenced by the immobilization period [47, 51]. Therefore, our result for the control group demonstrates that such derivations acted as a whole during the motor act preparation; this way, the coherence increase indicates that, in order to execute the motor act, these derivations participated together to help the preparation, therefore working as a pre-stimulus and helping in the case of a future motor response [21, 52, 53].

### Theta band oscillations in sensorimotor integration

Theta band coherence increased in the P3/Pz derivations in both the control and experimental groups. Such finding shows that movement control is distributed among the neuron populations which codify movement information; that is, the neural network modifies sensory information, turning it into an appropriate command for the motor system [54–56]. This way, sensorimotor activities occurred thanks to the integration of proprioceptive activities, in order to produce an appropriate motor response [57]. Additionally, we have observed that, apart from the functional relationship between the P3/Pz derivations with sensorimotor activity and motor act preparation [58–60], the fact of having greater coupling showed that these regions acted together, in order to facilitate the planning and organization of the sequence required to correctly execute the task [61], so that the motor act could be anticipated and selected [61] to be better executed [62].

When the theta band absolute power was investigated for the P3/P4 derivations, it could be observed that the sensory stimuli integration was modified by hand immobilization, therefore altering the relationship with the codification of the movements related to the task [63, 64]. Our results indicate greater coupling between the intra-hemispheric derivations, with less coupling for the P3-P4 derivations, meaning that the used intervention promoted inter-hemispheric coherence reduction in the parietal region. In addition to this, our findings can be related to the study by Catalan [65] and by Barany [66], who have defined the important role of the parietal cortex in the hand movement processing. The authors suggest that training promotes an involvement of these regions in motor program storing. Therefore, since our task did not involve complex movements nor previous training, theta coherence decreased for the P3/P4 derivations, showing that mechanisms of the sensorimotor integration associated with cognitive processing [15, 16, 20] and the sensory stimuli identification and codification [17] for the motor task planning underwent modifications [4, 31].

Theta band coherence also decreased in the T3/T4 derivations in the experimental group. Such alteration after the immobilization period (i.e. 48 hours) demonstrates that the temporal areas act as a multi-sensory integration center [67], that is, they are related to visual, auditory and somatosensory stimuli [68] and movement planning [69]. In their study, Balslev and colleagues asked the participants to reproduce the index finger movement of their left hand, imitating what was appearing on a screen [69]. They observed that the right temporal area was the most active during the task, therefore highlighting the importance of this region in sensory conflict situations. Despite our study not having directly examined sensory incongruence, theta coherence reduction was found between the hemispheres in the temporal region, therefore indicating that hand immobilization caused sensory conflict. In addition to this, the relationship between the temporal cortex and motor task was analyzed by Shannon and Bucker [70]. They have observed that, during the task-related memory, multi-sensory integration processes are solicited less, indicating that the temporal cortex is also participating less, in order for the neural network to transform sensory information into an appropriate command for the motor system. This way, the temporal region is associated more to memory management than to the motor act [71]. Therefore, we believe that, because of our task not demanding memory recovery, there was no need for greater coupling in the T3/T4 derivations.

## Conclusion

Our study aimed at analyzing the cortical changes occurring after 48 hours of hand immobilization. Our hypothesis was that the hand immobilization period would promote changes in the inter and intra-hemispheric coherence, and that the theta band coherence oscillations would provide information about the neural mechanisms for motor act execution. Therefore, we conclude that greater inter-hemispheric coupling occurs, except for the motor cortex, which showed theta band coherence intra-hemispheric alteration. In addition to this, motor tasks can be observed through theta band coherence. Considering this, we expect that other studies will also point out theta band as a parameter to investigate tasks involving motor acts. Here, we have some limitations due to small sample; nevertheless these results may contribute to a detailed assessment of involved neurophysiological mechanism in motor act execution.
